# Examining the levels of awareness, anxiety, and hope regarding global climate change among university students participating in activities at youth offices

**DOI:** 10.3389/fpsyg.2025.1655401

**Published:** 2025-11-19

**Authors:** Metin Yildiz

**Affiliations:** Faculty of Sport Sciences, Munzur University, Tunceli, Türkiye

**Keywords:** climate change, youth office, awareness, anxiety, hope

## Abstract

**Background:**

Climate change is one of the most significant issues of today. This study aims to investigate climate change awareness, anxiety, and hope levels among university students who participate in activities at youth offices and examine the relationship between them.

**Methods:**

The study population consisted of student members who were actively engaged in Youth Offices located within four public universities in the Eastern Anatolia Region of Türkiye. The study sample includes 881 volunteer participants, consisting of 345 (39.2%) male and 536 (60.8%) female students. The data were analyzed employing IBM SPSS 25. Statistical analyses included the independent samples *t*-test, one-way ANOVA, Tukey’s *post-hoc* test, and Pearson’s correlation coefficient.

**Results:**

The results indicate that university students have moderate levels of climate change awareness, anxiety, and hope. A positive correlation was observed, wherein higher awareness levels were associated with increased anxiety and hope. Male students demonstrated higher awareness and anxiety levels than female students. Among participants, students from 1. University exhibited the highest levels of climate change awareness, anxiety, and hope. Additionally, graduate students and individuals living in rural areas showed higher awareness and anxiety levels, whereas individuals with better economic conditions exhibited lower anxiety and hope levels.

**Conclusion:**

Given these results, it is recommended that universities develop and implement educational programs to enhance climate change awareness and extend these initiatives across campuses.

## 1 Introduction

Climate change, which affects every aspect of life, beginning with the environment in which we live, is among the most important public health threats nowadays. As reported in the Global Risks Report 2021, the top five probable global risks are extreme weather events, failure in climate change mitigation and adaptation efforts, human-induced environmental destruction, and biodiversity loss (World Economic Forum [WEF], 2021).

The impacts of climate change gradually increasing on a global scale pose a serious threat to all living organisms, from ecosystems to animals and humans (Intergovernmental Panel on Climate Change [IPCC], 2022). The severity of this threat has become increasingly evident through increasing temperatures and extreme weather events in recent years. Furthermore, psychology investigating human and animal behaviors has been focusing gradually more on the impacts of climate change on individuals and communities, highlighting that climate change is not only an environmental problem but also a psychological stressor (Clayton, 2020). Today, climate change is both a direct and indirect source of stress in various areas, and it is projected to lay the foundation for numerous stress factors on a global scale in the future (Mah et al., 2020). In this context, psychology science and psychologists are reported to play a pivotal role in helping individuals cope with climate-related stress and develop adaptive strategies for a healthy adjustment process (Heeren and Asmundson, 2023; Mah et al., 2020).

Given its detrimental impacts on human health and ecosystems, climate change has become a major global concern (Türkmen, 2021). Its effect on human health is anticipated to be the most significant global health challenge over the next decade (Anåker et al., 2021). Climate change refers to long-term alterations in weather patterns caused by human activities, which can lead to various health consequences for the global population (Amerson et al., 2022). Environmental phenomena driven by global climate change, including rising sea levels, increasing temperatures, extreme weather events, droughts, floods, and wildfires, can negatively influence human health and well-being (Dzurec, 2020; Rocque et al., 2021). Moreover, climate change is a multifaceted phenomenon with social, economic, political, geographical, ecological, and psychological ramifications (Abbasi and Nawaz, 2020). Recently, the impact of climate change on human health and healthcare systems has become an increasing concern (Ekici, 2022).

These effects can be categorized into environmental and socioeconomic dimensions. Environmentally, its consequences include desertification, biodiversity loss, and deforestation, while it socioeconomically impacts poverty levels, public health, livelihoods, and economic development (Ataklı and Kuran, 2022).

Managing the climate crisis and mitigating its effects should not be confined solely to scientific and technical solutions; rather, it must be transformed into a broader societal movement. In particular, raising awareness and mobilizing young individuals is a critical factor. As future decision-makers and the backbone of social movements, younger generations play a significant role in fighting against the environmental crisis (Hoffman, 2021).

As global climate patterns continue to change at an alarming rate, nations throughout the world are struggling to address the complex challenges caused by climate change (Kurnaz, 2023).

Türkiye was identified as one of the countries vulnerable to climate change in IPCC reports (Intergovernmental Panel on Climate Change [IPCC], 2021). According to the draft report published by the Turkish Grand National Assembly Climate Research Commission in August 2021, the intensity and frequency of extreme weather events in Türkiye have increased and are expected to further increase in the coming years (TBMM, 2021).

When examining the dictionary definition of “awareness,” it is described as “the state of being aware,” while being aware is defined as “having knowledge of things that need to be seen or understood, paying attention to a subject that requires comprehension” (TDK, 2022). Awareness is not only a skill that can be learned but also a process. In this process, individuals first begin to sense certain things. Over time, these sensations transform into knowledge, and knowledge leads to new understandings. Thus, awareness plays a significant role in adapting to global climate change (REC Türkiye, Bölgesel Çevre Merkezi., 2015).

Awareness of the factors contributing to climate change and the problems it causes is critical for fighting against these issues and developing solutions. Measuring climate change awareness is essential for identifying the steps to be taken in this struggle and for preparing action and education plans (Ataklı and Kuran, 2022). Since young people, in particular, will face the long-term effects of climate change more significantly, studies on this group become more important (Jürkenbeck et al., 2021).

At this point, universities can play a leading role in increasing environmental awareness and adopting sustainability policies. Universities can raise students’ consciousness about environmental issues through both theoretical and practical knowledge, encouraging them to take action (Lozano et al., 2019). Young people’s motivation to participate in the ecological transformation process may increase when projects promoting energy conservation, zero-waste policies, and green campus initiatives are implemented on university campuses (Deniz et al., 2021).

Turkish Language Association defines “anxiety” as “a thought that causes distress and worry” and “a feeling of tension that arises, often without a known cause, due to the anticipation of something bad happening” (TDK, 2024).

The effects of climate change on human health include physiological (allergies, heat-related illnesses, etc.), psychological (stress, anxiety, depression, etc.), and public health (increased violence and aggression, decreased social cohesion, etc.) dimensions (Karaman, 2022). In this regard, concerns about current and future environmental problems resulting from climate change are referred to as climate change anxiety (Clayton, 2020).

Young individuals report that their greatest anxiety about the climate crisis stems from its potential impact on their lives and futures. The climate crisis has numerous adverse effects, including economic uncertainties, food insecurity, and water crises. Additionally, this crisis exacerbates natural disasters, endangering human living spaces and triggering mass migrations on a global scale (Clayton and Karazsia, 2020).

The psychological effects of anxiety are also noteworthy. Specifically, “climate anxiety” has led many young people to experience hopelessness and pessimism about the future. This situation may influence their willingness to participate in environmental movements and support ecological transformation projects (Moser, 2020).

The Turkish Language Association defines “hope” as “a feeling arising from expectation” and “something anticipated or believed to happen” (TDK, 2023). In psychological literature, hope refers to an individual’s sense of expectation and confidence regarding uncertain yet potentially positive future circumstances. This feeling can enhance one’s motivation to achieve goals and strengthen the ability to cope with challenges (Snyder, 2002).

Young individuals’ hope in combating global climate change is often fueled by social movements and government policies. Effective steps taken by governments and international organizations can encourage individual participation (Hoffman, 2021).

In this context, it is critically necessary to determine and analyze the levels of climate change awareness, anxiety, and hope among university students, who are the future implementers. Studies on this subject will not only raise awareness among students but also shed light on relevant educational, practical, and academic studies. This study primarily aims to examine the climate change awareness, anxiety, and hope levels of university students participating in activities organized by youth offices and to explore the relationships among these variables. Additionally, another objective of this study is to examine whether young people’s levels of climate change awareness, anxiety, and hope are influenced by different demographic variables.

## 2 Materials and methods

### 2.1 Research pattern

This study employs a correlational survey model, a quantitative research method. This model is employed in quantitative studies to reveal the relationship between two or more variables (Büyüköztürk, 2015).

### 2.2 Study group/population sample

The study population consisted of student members who were actively engaged in Youth Offices located within four public universities in the Eastern Anatolia Region of Türkiye. Youth Offices are units established in areas with a high concentration of young people, serving as implementation centers of the “service-to-youth-at-their-doorstep” policy. Their primary objectives are to broaden the scope of youth activities and services, coordinate volunteering initiatives, and facilitate young people’s access to the projects of the Ministry of Youth and Sports of Türkiye.

To determine the required sample size, the G*Power software (version 3.1.9.2; Düsseldorf, Germany) was utilized. The results of the power analysis indicated that the study could be completed with 591 participants (effect size: 0.80; actual power: 0.89, taking Climate Change Anxiety as the outcome measure) (Özbay and Alcı, 2021). To minimize potential issues, survey forms were administered to 1,000 individuals using a simple random sampling method. However, due to exclusion criteria and incomplete responses, a total of 881 valid questionnaires were included in the analysis, comprising 345 (39.2%) male and 536 (60.8%) female participants.

### 2.3 Research process

Prior to the commencement of this study, the necessary permissions were obtained from the Munzur University Non-Interventional Research Ethics Committee. The data collection process was initiated in December 2024, with a focus on members employed within the Youth Offices of four state universities. The data were collected via a survey administered on a voluntary basis. Youth leaders responsible for conducting the survey were provided with the necessary instructions beforehand. They were also instructed to inform Youth Center members that the survey was for academic purposes, that there was no right or wrong answer, that their sincere responses would contribute to the research, and that their information would remain confidential (these statements were also included in the survey form). After this briefing, the members completed the survey form.

### 2.4 Data collection tools

The research data were collected using a survey form, which included a personal information form, the Global Climate Change Awareness Scale for University Students (GCCAS), the Climate Change Hope Scale (CCPHS), and the Climate Change Anxiety Scale (CCAS). The survey forms were administered both face-to-face and via Google Survey, ensuring data collection based on voluntary participation. Ethical approval to administer the scales and collect data was granted by the Non-Interventional Research Ethics Committee of Munzur University (Approval Date: 11/28/2024, Meeting No: 2024/10, Decision No: 01). Participation in this study was entirely voluntary.

**Personal Information Form (PIF)**: This form was designed by the researchers to collect demographic and background data that could be used as independent variables, including the Youth Office attended, sex, age, education level, class year, perceived level of well-being, the longest place of residence, and whether the participant had received any courses or training on climate change.

**Global Climate Change Awareness Scale for University Students (GCCAS)**: Developed by Deniz et al. (2021), this scale measures university students’ awareness of global climate change. It consists of 21 items and four subdimensions: (1) effects on natural and human environments (ENHE) (9 items), (2) awareness of global organizations and agreements (AGOA) (6 items), (3) causes of climate change (CCC) (3 items), and (4) relationship with energy consumption (REC) (3 items). Each item is rated on a 5-point Likert scale: 1 = Not aware at all, 2 = Not aware, 3 = Neutral, 4 = Aware, 5 = Fully aware. The scale does not contain any reverse-coded items. Scores range between 21 and 105, and higher scores indicate a higher level of awareness of global climate change. Furthermore, when the total scale and subdimension scores are divided by the number of items, awareness levels are categorized as follows: 1.00–2.33 (low), 2.34–3.66 (moderate), and 3.67–5.00 (high). The reliability coefficient (Cronbach’s Alpha) for the original scale was 0.826, while it was calculated to be 0.907 in the present study.

**Climate Change Anxiety Scale (CCAS):** It consists of 10 items and was developed by Stewart (2021) and adapted into Turkish by Özbay and Alcı (2021). The factor analysis results indicate that the Turkish adaptation of the scale remains valid as a unidimensional construct. The reliability coefficient (Cronbach’s alpha) of the scale was calculated to be 0.980. The scale was reported to be a valid and reliable instrument for measuring climate change anxiety among university students in Türkiye (Özbay and Alcı, 2021). In this study, the reliability coefficient (Cronbach’s alpha) of the CCAS was calculated as 0.855.

Responses to the CCAS were assessed based on the total scores assigned to each item. The items in the scale are rated on a five-point Likert scale: “Strongly Disagree,” “Disagree,” “Neutral,” “Agree,” and “Strongly Agree.” The minimum possible score on the scale is 10, while the maximum is 50. A score closer to 50 indicates higher levels of climate change anxiety, whereas a score closer to 10 signifies lower anxiety levels. The scale does not include any reverse-coded items.

**Climate Change Hope Scale (CCHS):** It was developed by Li and Monroe (2018) to measure hope regarding climate change mitigation and was adapted into Turkish by Gezer and İlhan (2021). This five-point Likert-type scale consists of 11 items and three subdimensions: Individual (items 1, 2, and 3), Societal (items 4, 5, 6, 7, and 8), and Despair (items 9, 10, and 11). The response format ranges from “Strongly Agree” (5) to “Strongly Disagree” (1). The internal consistency coefficients (Cronbach’s alpha) for the subdimensions were as follows: 0.56 for the Individual subdimension, 0.65 for the Societal subdimension, and 0.62 for the Despair subdimension. The overall Cronbach’s alpha for the scale was 0.74. Additionally, composite reliability coefficients ranged from 0.58 to 0.87. In this study, the reliability coefficient (Cronbach’s alpha) of the CCHS was calculated as 0.603.

### 2.5 Data analysis/statistical method

The data were analyzed employing IBM SPSS 25. The reliability of the scales was assessed employing Cronbach’s alpha coefficient, with values of 0.907 for GCCAS, 0.603 for CCHS, and 0.855 for CCAS. Frequencies and percentages were presented for categorical variables, while means and standard deviations were provided for continuous variables. The independent samples *t*-test was used to compare quantitative variables between two-category qualitative variables. For comparisons involving qualitative variables with more than two categories, one-way ANOVA was employed. If a significant difference was detected through one-way ANOVA, pairwise comparisons were conducted using Tukey’s test. The relationships between two continuous variables were examined using Pearson’s correlation coefficient. The Type I error rate was set at 0.05 for this study.

## 3 Results

In this research conducted with the Youth Center members participating in the activities in the youth offices of the universities affiliated to the Youth Centers, the relationship between the awareness, anxiety and hope levels of the university students participating in the activities in the youth offices of the universities toward global climate change was determined and examined in terms of independent variables and the findings obtained in line with the research questions were presented in the relevant titles.

The table presents age distribution, university affiliation, sex, educational level, academic year (The preparatory class is a class that both develops foreign language skills and facilitates adaptation to school), primary place of residence, and self-perceived economic income level compared to the general population. In addition, participants’ prior education or coursework on climate change is reported. These variables were considered as potential determinants in the analysis of participants’ perceptions, knowledge, and attitudes toward climate change (Table 1).

**TABLE 1:** Demographic characteristics of participants.

Descriptive	f (%)
Age $\bar{x}\pm S\,D$	21.88 ± 2.85
**Youth office**	
1. University youth office	235 (26.7)
2. University youth office	202 (22.9)
3. University youth office	228 (25.9)
4. University youth office	216 (24.5)
**Sex**	
Male	345 (39.2)
Female	536 (60.8)
**Educational level**	
College	148 (16.8)
Undergraduate	650 (73.8)
Postgraduate	83 (9.4)
**Year**	
Preliminary	85 (9.6)
1st year	243 (27.6)
2nd year	312 (35.4)
3rd year	136 (15.4)
4th year	105 (11.9)
**Place where you’ve spent most of your life**	
Metropolitan	171 (19.4)
City	409 (46.4)
District	213 (24.2)
Village	88 (10)
**Self-perceived economic income level in comparison to the general population**	
Very good: we can spend money as we wish	68 (7.7)
Good: we do not have difficulty meeting our needs	211 (24)
Moderate: we can only meet our needs	356 (40.4)
Poor: we are unable to fully meet our needs	134 (15.2)
Very poor: we struggle to meet our needs	112 (12.7)
**Previous education or coursework on climate change**	
Yes	246 (27.9)
No	635 (72.1)

Given the data presented in Table 2, participants’ global climate change awareness, level of hope, and climate change anxiety were determined to be at a moderate level. The mean score for the GCCAS was 3.39 ± 0.85, indicating a moderate level of awareness regarding climate change issues. The mean score for the CCHS was 3.33 ± 0.54, suggesting that while participants exhibited hope and a willingness to learn about combating climate change, this tendency could be further strengthened. The CCAS yielded a mean score of 3.31 ± 0.79, demonstrating that participants experienced a certain degree of anxiety concerning climate change; however, this anxiety was not at a high level. Overall, these results suggest that participants have a moderate level of awareness and interest in climate change. However, further efforts are needed to enhance awareness and encourage proactive engagement in climate action initiatives.

**TABLE 2:** Mean scores of the scales.

Scales	$\bar{\text{x}}\,\text{S}\,D$
GCCAS_mean_	3.39 ± 0.85
ENHE	3.51 ± 0.96
AGOA	3.25 ± 1.02
CCC	3.18 ± 1.13
REC	3.52 ± 1.07
CCHS_mean_	3.33 ± 0.54
CCAS_mean_	3.31 ± 0.79
Individual	3.56 ± 0.97
Societal	3.49 ± 0.87
Despair	2.83 ± 1.03

Examining Table 3, the comparisons between demographic variables and scale scores reveal the following findings. Participants from the 1. University Youth Office had the highest GCCAS scores, whereas participants from the 4. University Youth Office had the lowest (*p* < 0.001). Similarly, participants from the 1. University Youth Office exhibited the highest CCHS scores, while those from 3. University and 4. University Youth Offices had the lowest scores (*p* < 0.001). Regarding CCAS scores, 1. University Youth Office participants scored significantly higher than participants from other Youth Offices (*p* < 0.001). In terms of sex differences, male participants reported significantly higher GCCAS (*p* = 0.005) and CCAS (*p* = 0.009) scores than female participants. However, no significant difference was found between male and female participants regarding CCHS scores (*p* = 0.169). When examining differences by educational level, participants at the graduate level had significantly higher CCAS scores than those at the associate degree level. However, there was no significant difference in GCCAS (*p* = 0.142) or CCHS (*p* = 0.235) scores across educational levels. Comparisons across academic years indicated that preparatory and fourth-year students had significantly higher GCCAS scores than first-year students (*p* = 0.001). However, no significant difference was found in CCAS scores (*p* = 0.581). While a one-way ANOVA test suggested a significant difference in CCHS scores across academic years (*p* = 0.010), pairwise comparisons using the Tukey test did not reveal any distinct category differences. Regarding residential background, participants who had spent most of their lives in rural areas had significantly higher GCCAS scores than other participants (*p* < 0.001). Similarly, those who had spent the majority of their lives in rural areas exhibited higher CCAS scores compared to participants who had primarily lived in metropolitan or urban areas (*p* = 0.002). However, no significant difference was found in CCHS scores across residential location categories (*p* = 0.164). Economic status comparisons indicated that participants who reported having a very good economic situation had the lowest GCCAS scores (*p* < 0.001). Moreover, those who described their economic situation as very good had significantly lower CCHS scores (*p* = 0.006). Furthermore, participants who rated their economic status as very good or very poor had lower CCAS scores than other participants (*p* < 0.001). Finally, participants who had received education on climate change exhibited significantly higher GCCAS scores (*p* = 0.007). However, no significant differences by climate change education status were observed in CCHS (*p* = 0.070) or CCAS (*p* = 0.251) scores.

**TABLE 3:** Comparisons between demographic variables and scale scores.

Descriptive	GCCAS_mean_	CCHS_mean_	CCAS_mean_
**Center**			
1. University youth office (A)	3.85 ± 0.64	3.56 ± 0.47	3.64 ± 0.67
2. University youth office (B)	3.42 ± 0.7	3.43 ± 0.53	3.18 ± 0.76
3. University youth Office (C)	3.23 ± 1.17	3.17 ± 0.56	3.19 ± 1.01
4. University youth office (D)	3.01 ± 0.45	3.15 ± 0.48	3.19 ± 0.52
F	46.679	35.11	20.02
η	0.138	0.107	0.064
p	<0.001*	<0.001*	<0.001*
Difference	A > B, C > D	A > B > C,D	A > B, C, D
**Gender**			
Male	3.48 ± 0.74	3.36 ± 0.53	3.39 ± 0.73
Female	3.33 ± 0.91	3.31 ± 0.55	3.25 ± 0.82
t	2.79	1.376	2.615
η	0.177	0.092	0.178
p	0.005*	0.169	0.009*
**Educational level**			
College (A)	3.27 ± 0.68	3.3 ± 0.54	3.16 ± 0.69
Undergraduate (B)	3.4 ± 0.88	3.32 ± 0.55	3.32 ± 0.8
Postgraduate (C)	3.48 ± 0.9	3.42 ± 0.48	3.48 ± 0.87
F	1.955	1.449	4.82
η	0.004	0.003	0.011
p	0.142	0.235	0.008*
Difference	–	–	C > A
**Year**			
Preliminary	3.5 ± 0.8	3.36 ± 0.54	3.41 ± 0.75
1st year	3.2 ± 0.96	3.25 ± 0.53	3.25 ± 0.85
2nd year	3.44 ± 0.75	3.41 ± 0.55	3.31 ± 0.79
3rd year	3.4 ± 0.84	3.31 ± 0.53	3.35 ± 0.72
4th year	3.56 ± 0.85	3.26 ± 0.53	3.29 ± 0.75
F	4.924	3.360	0.716
η	0.022	0.015	0.003
p	0.001*	0.010*	0.581
Difference	p, 4 > 1	–	–
**Place of residence**			
Metropolitan (A)	3.33 ± 0.73	3.39 ± 0.48	3.29 ± 0.68
City (B)	3.28 ± 0.92	3.29 ± 0.56	3.22 ± 0.89
District (C)	3.49 ± 0.81	3.35 ± 0.54	3.4 ± 0.7
Village (D)	3.73 ± 0.67	3.33 ± 0.55	3.53 ± 0.63
F	8.353	1.708	4.974
η	0.028	0.006	0.017
p	<0.001*	0.164	0.002*
Difference	D > A.B.C	–	D > A.B
**Economic status**			
Very good: we can spend money as we wish (A)	2.65 ± 1.15	3.1 ± 0.59	2.85 ± 1.13
Good: we do not have difficulty meeting our needs (B)	3.41 ± 0.98	3.37 ± 0.58	3.34 ± 0.81
Moderate: we can only meet our needs (C)	3.41 ± 0.67	3.34 ± 0.49	3.39 ± 0.67
Poor: we are unable to fully meet our needs (D)	3.58 ± 0.69	3.36 ± 0.53	3.51 ± 0.71
Very poor: we struggle to meet our needs (E)	3.49 ± 0.84	3.28 ± 0.57	3.01 ± 0.76
F	16.184	3.687	13.992
η	0.069	0.017	0.060
P	<0.001*	0.006*	<0.001*
Difference	B.C.D.E > A	B.C.D > A	B.C.D > A.E
**Previous education or coursework on climate change**			
Yes	3.5 ± 0.73	3.38 ± 0.54	3.36 ± 0.76
No	3.34 ± 0.89	3.31 ± 0.54	3.29 ± 0.8
t	2.701	1.814	1.148
η	0.189	0.130	0.001
p	0.007*	0.070	0.251

*p < 0.001.

As seen in Table 4, the analysis of comparisons between age and scale scores reveals a very weak negative correlation between age and GCCAS scores (*p* < 0.001; *r* = −0.137). No significant relationship was found between age and CCHS (*p* = 0.057) or CCAS (*p* = 0.051). A weak positive correlation was identified between GCCAS and CCHS (*p* < 0.001; *r* = 0.398), whereas a moderate positive correlation was determined between GCCAS and CCAS (*p* < 0.001; *r* = 0.496). Additionally, a weak positive correlation was found between CCHS and CCAS (*p* < 0.001; *r* = 0.366). Overall, the results suggest that age does not have a significant impact on scale scores; however, the scales themselves exhibit significant and positive interrelationships.

**TABLE 4:** Comparisons between age and scale scores.

Descriptive		GCCAS_mean_	CCHS_mean_	CCAS_mean_
Age	r	−0.137*	−0.064	−0.066
	p	<0.001	0.057	0.051
GCCAS_mean_	r	1	0.398*	0.496*
	p		<0.001	<0.001
CCHS_mean_	r		1	0.366*
	p			<0.001

*p < 0.001.

## 4 Discussion and conclusion

In the study, the awareness, anxiety and hope levels of university students participating in activities in the youth offices of universities toward global climate change and the relationship between them were investigated. Given the data presented in Table 2, the university students participating in activities at youth offices exhibit moderate levels of global climate change awareness, hope, and anxiety. A study carried out by Fertelli (2023) similarly reported that participants’ climate change awareness, concerns, and hope were at a moderate level. These findings suggest that the results of this study align with those reported by Fertelli. Additionally, İlaslan and Şahin Orak (2023) reported students’ awareness of climate change to be at a moderate level (3.43 ± 0.69), whereas their climate change-related concerns were at a low level (33.10 ± 8.08). These results partially support the results of the present study.

In this study, the participants’ mean score on the GCCAS was found to be 3.39 ± 0.85, indicating a moderate level of climate change awareness. Similarly, the mean score on the CCHS was 3.33 ± 0.54, suggesting that participants are hopeful and open to learning about climate change mitigation, although this tendency could be further enhanced. On the other hand, the mean CCAS score was calculated to be 3.31 ± 0.79, indicating that participants experience a certain level of anxiety regarding climate change, but not at high levels. Overall, it can be stated that participants exhibit moderate levels of awareness, hope, and anxiety concerning climate change, yet there is a need for further support in raising awareness and fostering action on this issue. Tümer et al. (2024) reported that the mean climate change awareness score of participants was 85.27 ± 6.70, indicating a good level of awareness. In the same study, the mean CCAS score was 32.45 ± 7.28, while the mean CCHS score was 38.14 ± 5.39. These findings suggest that participants’ levels of concern and hope regarding climate change were moderate. Furthermore, a significant positive relationship was identified between awareness, concern, and hope (Tümer et al., 2024), which aligns with the findings achieved in this study. Additionally, Duman et al. (2024) revealed a significant negative relationship between climate change anxiety and psychological resilience and hope, whereas a significant positive relationship was found between psychological resilience and hope (Duman et al., 2024).

The positive correlations identified in this study between awareness, anxiety, and hope highlight the dual psychological consequences of increased awareness. On the one hand, greater awareness may strengthen individuals’ sense of agency and foster constructive engagement, thereby reinforcing hope. This process is likely mediated by mechanisms such as environmental self-efficacy, collective efficacy beliefs, and perceived social support. On the other hand, higher awareness may also heighten perceptions of existential threat, particularly among individuals with low perceived control, thereby fueling dysfunctional anxiety. Thus, the direction of the impact of awareness on psychological outcomes appears to depend on factors such as coping strategies, resilience, and socio-cultural norms. These findings underline the necessity for future research to explore these mediating mechanisms more directly, for instance through mediation analysis, structural equation modeling, or qualitative inquiry, in order to clarify under which conditions awareness promotes adaptive hope or maladaptive anxiety (Clayton, 2020; Ojala, 2012).

It is noteworthy that the internal consistency of the Climate Change Hope Scale in this study was relatively low (Cronbach’s α = 0.603). While this value approaches the minimum acceptable threshold in exploratory research, it raises concerns regarding the construct validity and interpretability of the results related to hope. A coefficient at this level suggests that the items may not have been sufficiently homogeneous in capturing the underlying construct. Consequently, the findings concerning hope should be interpreted with caution. One possible explanation is that hope is a multidimensional concept encompassing individual, societal, and despair-related dimensions, which may not have been adequately represented in a single composite score. For future research, analyzing these sub-dimensions separately may provide a more nuanced understanding of how hope operates in the context of climate change. Alternatively, refining the measurement tool or employing other validated instruments with stronger psychometric properties would help enhance the robustness of conclusions in this domain.

From a developmental perspective, it is also important to recognize that university students represent more than a convenient sample; they are situated within the life stage of emerging adulthood (Arnett, 2000), which is characterized by identity exploration, social responsibility, and emotional vulnerability. These characteristics may partly explain why climate-related threats evoke both anxiety and hope within this group. Furthermore, self-determination theory (Ryan and Deci, 2000) provides a useful motivational lens to interpret our findings: when students’ basic psychological needs for autonomy, competence, and relatedness are supported, climate anxiety may be transformed into constructive engagement and sustained hope, whereas unmet needs may contribute to avoidance or maladaptive distress. Recent research has shown that environmental concern among emerging adults plays a critical role in shaping future thinking and sustainable career aspirations (La Rosa and Zammitti, 2025), reinforcing the relevance of developmental and motivational frameworks for interpreting climate-related psychological responses. Integrating these perspectives suggests that interventions in higher education should not only raise awareness but also support students’ developmental and motivational needs, thereby enhancing resilience and fostering meaningful engagement with climate action.

In addition to climate-related concerns, prior research indicates that university students frequently display heightened emotional reactivity in other domains of daily life. For instance, Mammadzade et al. (2020) demonstrated that students’ social media attitudes were significantly associated with their levels of anger. This finding supports the idea that young adults, situated in the emotionally vulnerable stage of emerging adulthood, may react strongly not only to ecological threats but also to digital and social stimuli. Integrating such evidence underscores that climate anxiety should be interpreted within the broader spectrum of students’ emotional experiences, further highlighting the need for universities to provide psychosocial support that addresses multiple sources of stress and emotional regulation challenges.

Although several group comparisons yielded statistically significant results, the effect sizes calculated with Cohen’s *d* were negligible across all *t*-tests. This suggests that, while differences between groups exist, their practical significance is limited. Consequently, these findings highlight that broader psychosocial and institutional factors may play a more meaningful role than demographic variables in shaping students’ climate-related attitudes and emotions.

The present findings also raise important questions regarding the role of Youth Offices in higher education settings. While these units appear to provide students with extracurricular opportunities for engagement and activism, it remains unclear whether they function merely as activity hubs or as institutional support channels that can foster students’ emotional resilience in the face of climate-related concerns. Given the growing recognition that climate anxiety can affect students’ learning, well-being, and civic participation, universities should move beyond awareness-raising efforts and develop comprehensive support systems. Evidence-based interventions such as psychoeducational workshops, curricular innovations integrating sustainability and mental health perspectives, and peer-support initiatives could help transform anxiety into constructive engagement while simultaneously reinforcing hope. Embedding such interventions within Youth Offices or in collaboration with them would position these structures not only as platforms for activism but also as critical spaces for psychosocial support, thereby enhancing both individual and collective coping capacities.

As seen in Table 3, significant findings were obtained from the comparisons between demographic variables and scale scores. Participants from 1. University’s Youth Office had the highest scores on the GCCAS, whereas participants from 4. University’s Youth Office had the lowest ones (*p* < 0.001). This finding indicates that participants from 1. University exhibit a higher level of climate change awareness. Similarly, regarding the CCHS scores, 1. University’s Youth Office participants had the highest scores, whereas participants from 3. University and 4. University’s Youth Offices had the lowest ones (*p* < 0.001). This result suggests that participants from 1. University are more hopeful about combating climate change, whereas participants from other universities exhibit lower levels of hope in this regard. In terms of CCAS scores, participants from 1. University’s Youth Office scored significantly higher than participants from other universities (*p* < 0.001). This finding implies that 1. University participants experience a higher level of anxiety regarding climate change. This trend can be interpreted as an indication that anxiety levels may also rise as awareness levels increase. Furthermore, the differences in climate change awareness, hope, and anxiety levels among participants from various universities highlight the necessity of disseminating educational and awareness-raising initiatives on climate change in a more equitable and effective manner across universities.

Table 3 indicates that when comparing scale scores by demographic variables, male participants exhibited significantly higher scores than female participants on both the GCCAS (*p* = 0.005) and the CCAS (*p* = 0.009). However, no significant difference was found between male and female participants in terms of CCHS scores (*p* = 0.169), suggesting that sex influences climate change awareness and anxiety levels, but not the level of hope for preventing climate change. The higher GCCAS and CCAS scores among male participants indicate that they may have a higher level of awareness of and anxiety about climate change. However, the underlying reasons for this discrepancy require further in-depth investigation.

The finding that male students reported higher levels of both awareness and anxiety stands in partial contrast to a substantial body of climate psychology literature, which typically suggests that women tend to experience stronger eco-anxiety and greater concern about environmental risks (Clayton, 2020; Gülsoy and Korkmaz, 2020; Yörük and Akpınar, 2023). This inconsistency may be explained by several contextual and methodological factors. First, cultural gender norms in Türkiye may shape how men and women perceive and report climate-related concerns. Men, who are often expected to display responsibility and knowledge in public spheres, may report higher awareness and anxiety as a reflection of socially reinforced roles. Second, social desirability bias could have influenced self-report measures, leading female participants to underreport their anxiety to avoid appearing “overly emotional” or “pessimistic,” while men may have emphasized their awareness as a marker of social competence. Finally, the finding may reflect context-specific exposure: if male students in Youth Offices are more engaged in outdoor activities, policy discussions, or environmental projects, they may have greater exposure to information that heightens both awareness and anxiety. Future studies employing mixed-method approaches including qualitative interviews could provide a deeper understanding of how gender norms and reporting tendencies influence self-assessments of climate change-related emotions.

The literature presents both partially consistent and conflicting findings with these results. For instance, Tümer et al. (2024) found no significant difference in the mean scores of the GCCAS, CCAS, and CCHS by age group and sex (Tümer et al., 2024). This result partially contradicts the sex differences found in the present study. Conversely, Yörük and Akpınar (2023) determined that female students exhibited higher levels of awareness regarding global climate change in comparison to male students (Yörük and Akpınar, 2023). This finding contradicts the present study’s result that male participants had higher awareness scores. Similarly, Gülsoy and Korkmaz (2020) reported that female students placed greater importance on global warming and climate change and demonstrated higher levels of consciousness on these issues when compared to male students (Gülsoy and Korkmaz, 2020). These results indicate that the influence of sex on attitudes toward climate change is complex and multidimensional.

Comparisons across different education levels revealed that participants at the graduate level had significantly higher scores on the CCAS when compared to those at the associate degree level. However, no significant differences were determined between education level categories in terms of GCCAS (*p* = 0.142) or the CCHS (*p* = 0.235) scores. These findings suggest that individuals at the graduate education level exhibit a higher level of awareness regarding global climate change, whereas anxiety and hope levels appear to be independent of education level.

Comparisons across academic years indicated that preparatory-year and senior-year students had significantly higher scores on the GCCAS when compared to first-year students (*p* = 0.001). This finding suggests that even though preparatory-year students have not yet started their undergraduate studies, they experience higher levels of climate change anxiety than first-year students. Furthermore, the fact that senior-year students also had higher anxiety scores than first-year students implies that anxiety levels concerning climate change may fluctuate at different stages of the educational process. In a study carried out in 2020, Clayton and Karazsia noted that climate change anxiety varies depending on individuals’ life stages and the contexts in which they are situated (Clayton and Karazsia, 2020). On the other hand, no significant difference was found among academic years regarding scores on the CCAS (*p* = 0.581). This result suggests that students’ awareness levels of climate change remain relatively consistent regardless of their academic year. The one-way ANOVA test results for the CCHS scores indicated a significant difference among academic years (*p* = 0.010). However, pairwise comparisons using Tukey’s test did not identify specific academic years that differed significantly from one another. This finding suggests that while hope levels regarding climate change mitigation do not exhibit a distinct pattern across academic years, there is an overall heterogeneity among student groups.

Comparisons based on the variable of the longest place of residence revealed that participants who had spent most of their lives in a village had significantly higher scores on the GCCAS in comparison to other participants (*p* < 0.001). This finding suggests that individuals living in a village may experience higher levels of anxiety due to their more direct exposure to the effects of climate change or their closer connection with the natural environment. Reser et al. (2012) noted that individuals residing in rural regions are more likely to witness the impacts of climate change on agriculture, water resources, and ecosystems, which may contribute to increased anxiety levels (Reser et al., 2012). The findings of that study are consistent with the results achieved in the present research.

Additionally, it was found that participants who had spent most of their lives in rural areas had significantly higher scores on the CCAS in comparison to those who had lived primarily in metropolitan or urban areas (*p* = 0.002). This finding suggests that individuals residing in rural areas may develop a higher level of awareness regarding climate change due to their closer interaction with the natural environment. Milfont (2012) emphasized that individuals living in rural regions are more sensitive to environmental changes, which in turn increases their level of awareness (Milfont, 2012). The results of that study are also consistent with the results achieved in the present study.

On the other hand, no significant difference was found between settlement categories in terms of scores on the CCHS (*p* = 0.164). This result indicates that levels of hope regarding the mitigation of climate change are experienced similarly, regardless of individuals’ places of residence. Ojala (2012) suggested that levels of hope are more influenced by factors such as personal beliefs, motivations, and social support rather than by geographical location (Ojala, 2012).

Comparisons based on economic status categories revealed that participants who identified their economic status as “very good” had the lowest scores on the CCAS (*p* < 0.001). This finding suggests that individuals in a better economic position experience lower levels of climate change anxiety. Economic security may contribute to reduced anxiety about environmental threats. Gifford and Comeau reported that individuals with a higher level of economic security exhibit lower levels of concern regarding environmental risks, which in turn reduces their anxiety levels (Gifford and Comeau, 2011).

Additionally, it was found that participants who rated their economic status as “very good” had lower scores on the CCHS (*p* = 0.006), suggesting that individuals in a better economic position exhibit lower levels of hope regarding climate change prevention. This may indicate that economic prosperity reduces individuals’ motivation to address environmental issues or leads them to perceive these issues as less urgent. Ojala (2012) noted that individuals with a better economic standing may experience lower levels of hope regarding environmental problems, as they may perceive these issues as less threatening to their own lives.

On the other hand, participants who rated their economic status as either “very good” or “very poor” had lower scores on the CCAS compared to other participants (*p* < 0.001). This result indicates that both individuals with very high economic status and those with very low economic status tend to have lower levels of climate change awareness. This suggests that economic prosperity may make individuals less sensitive to environmental problems, while economic hardship may also reduce their level of environmental awareness. Scannell and Gifford (2013) found that economic difficulties diminish individuals’ capacity to focus on environmental issues.

Furthermore, it was found that participants who had received education on climate change had higher scores on the GCCAS (*p* = 0.007); however, there were no significant differences in their CCHS (*p* = 0.070) and CCAS (*p* = 0.251) scores. Tümer et al. (2024) reported no significant differences in the total scores on GCCAS, CCAS, and CCHS based on whether individuals had received climate change education. The results achieved in the present study align with those reported by Tümer et al. (2024), supporting the consistency of the results.

This study also examined the relationships between participants’ ages and their scale scores. The relationship between age and GCCAS scores indicates a very weak negative correlation (*p* < 0.001; *r* = −0.137). This indicates that climate change awareness levels tend to slightly decrease as age increases. However, given the very weak nature of this relationship, it cannot be concluded that age has a significant impact on climate change awareness. Relationship between age and CCHS and CCAS indicates no significant relationship between age and CCHS (*p* = 0.057) or between age and CCAS (*p* = 0.051). In other words, there was no significant change in hope or anxiety levels as participants’ ages increased. Relationships between GCCAS, CCHS, and CCAS indicate a weak positive correlation between GCCAS and CCHS (*p* < 0.001; *r* = 0.398), suggesting that individuals’ hope for preventing climate change also tends to increase slightly as climate change awareness increases. Additionally, a moderate positive correlation was found between GCCAS and CCAS (*p* < 0.001; *r* = 0.496), indicating that climate change anxiety also tends to increase at a moderate level as climate change awareness increases. The relationship between CCHS and CCAS suggests a weak positive correlation (*p* < 0.001; *r* = 0.366), suggesting that anxiety levels also tend to rise slightly as hope for preventing climate change increases.

This study has several limitations that should be acknowledged. First, the research employed a cross-sectional survey design, which restricts the ability to establish causal inferences between climate change awareness, anxiety, and hope. Longitudinal or experimental designs would provide stronger evidence regarding the directionality of these relationships. Second, the sample consisted of university students actively participating in Youth Offices from only four public universities in Eastern Anatolia, Türkiye. While this group provides valuable insights, the findings may not be generalizable to all university students in Türkiye or other cultural and geographical contexts. Third, self-reported questionnaires were the sole data collection method, which may have introduced response and social desirability biases. Fourth, although reliable and valid instruments were used, the relatively low internal consistency of the Climate Change Hope Scale (Cronbach’s α = 0.603) suggests that further refinement or complementary qualitative measures could strengthen the assessment of hope. Fifth, despite efforts to ensure diversity within the sample, certain demographic subgroups (e.g., postgraduate students, participants from rural areas) were underrepresented, potentially limiting statistical power in subgroup analyses. Finally, contextual factors such as regional climate experiences, media exposure, and political or institutional influences were not assessed, though these may significantly shape students’ perceptions and attitudes toward climate change. Future studies should employ mixed-method designs, include broader and more diverse populations, and examine longitudinal changes in climate change awareness, anxiety, and hope. Additionally, integrating qualitative approaches could provide deeper insights into the underlying mechanisms and lived experiences of young individuals in relation to climate change.

## 5 Conclusion

The findings of this study highlight that university students participating in youth office activities exhibit moderate levels of climate change awareness, hope, and anxiety, which aligns with or partially supports prior studies in the literature. Considering these results, several implications can be drawn:

- Educational Interventions
- The moderate awareness levels identified suggest the necessity of integrating climate change education into the curricula of higher education institutions.
- Interdisciplinary courses that combine environmental sciences, psychology, and social sciences could enhance students’ capacity to understand and act upon climate change.
- Psychological and Social Support
- The coexistence of moderate levels of hope and anxiety highlights the need for interventions aimed at transforming climate-related anxiety into constructive engagement.
- Universities could offer counseling programs, peer-support groups, and climate action workshops to balance anxiety with hopeful, proactive attitudes.
- Equity Across Institutions and Demographics
- Differences across universities, sex, academic years, and socioeconomic groups underline the need for tailored interventions.
- Policy-makers and educators should ensure that awareness-raising initiatives are implemented equitably across different institutions and student populations, particularly in regions with limited resources.
- Policy and Practice Implications
- Youth offices represent an effective platform for cultivating climate literacy and resilience. Expanding such initiatives could serve as a strategic model for other universities.
- Collaboration between universities, local governments, and NGOs may enhance the dissemination and impact of climate change education.
- Future Research
- Longitudinal studies are needed to examine how climate change awareness, hope, and anxiety evolve over time, particularly as students progress through their academic careers.
- Further investigation into the causal mechanisms underlying sex differences and economic disparities in awareness and anxiety levels would contribute to a deeper understanding of climate change attitudes. Experimental and intervention-based research could test the effectiveness of different educational and psychological support programs in enhancing awareness while mitigating excessive anxiety.

## Data Availability

The datasets presented in this study can be found in online repositories. The names of the repository/repositories and accession number(s) can be found in the article/supplementary material.
